# 4-[Bis(4-fluorophenyl)methyl]piperazin-1-ium 2-(2-phenylethyl)benzoate

**DOI:** 10.1107/S160053681103902X

**Published:** 2011-09-30

**Authors:** Richard Betz, Thomas Gerber, Eric Hosten, Alaloor S. Dayananda, Hemmige S. Yathirajan

**Affiliations:** a Nelson Mandela Metropolitan University, Summerstrand Campus, Department of Chemistry, University Way, Summerstrand, PO Box 77000, Port Elizabeth, 6031, South Africa; b University of Mysore, Department of Studies in Chemistry, Manasagangotri, Mysore 570 006, India

## Abstract

The asymmetric unit of the title salt, C_17_H_19_F_2_N_2_^+.^C_15_H_13_O_2_^−^, derived from a 1,4-diaza­cyclo­hexane derivative and a carb­oxy­lic acid, contains two formula units. The cation is protonated at the secondary amine functionality. The six-membered heterocycles adopt chair conformations. The fluorophenyl rings in the two cations make dihedral angles of 77.21 (19) and 78.8 (2)° while the aromatic rings in the anions enclose angles of 69.5 (2) and 69.9 (2)°. In the crystal, classical N—H⋯O hydrogen bonds as well as C—H⋯F and C—H⋯O contacts connect the entities into layers parallel to *ac*.

## Related literature

For the biological activity of piperazines, see: Brockunier *et al.* (2004 ▶); Bogatcheva *et al.* (2006 ▶). For related structures, see: Anilkumar *et al.* (2005 ▶); Betz *et al.* (2011 ▶); Fun *et al.* (2011 ▶); Jasinski *et al.* (2010 ▶, 2011 ▶); Dutkiewicz *et al.* (2011 ▶). For graph-set analysis of hydrogen bonds, see: Etter *et al.* (1990 ▶); Bernstein *et al.* (1995 ▶). For puckering analysis, see: Cremer & Pople (1975 ▶).
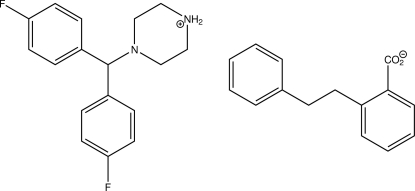


## Experimental

### 

#### Crystal data

C_17_H_19_F_2_N_2_^+^·C_15_H_13_O_2_^−^
*M* *_r_* = 514.60
Monoclinic, 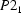

*a* = 8.2330 (2) Å
*b* = 35.5366 (10) Å
*c* = 10.1505 (3) Å
β = 112.925 (1)°
*V* = 2735.19 (13) Å^3^
*Z* = 4
Mo *K*α radiation
μ = 0.09 mm^−1^
*T* = 200 K
0.52 × 0.34 × 0.31 mm

#### Data collection

Bruker APEXII CCD diffractometer
24852 measured reflections
6889 independent reflections
6401 reflections with *I* > 2σ(*I*)
*R*_int_ = 0.033

#### Refinement

*R*[*F* ^2^ > 2σ(*F* ^2^)] = 0.050
*wR*(*F* ^2^) = 0.129
*S* = 1.07
6889 reflections
701 parameters
1 restraint
H atoms treated by a mixture of independent and constrained refinement
Δρ_max_ = 0.29 e Å^−3^
Δρ_min_ = −0.21 e Å^−3^

### 

Data collection: *APEX2* (Bruker, 2010 ▶); cell refinement: *SAINT* (Bruker, 2010 ▶); data reduction: *SAINT*; program(s) used to solve structure: *SHELXS97* (Sheldrick, 2008 ▶); program(s) used to refine structure: *SHELXL97* (Sheldrick, 2008 ▶); molecular graphics: *ORTEPIII* (Farrugia, 1997 ▶) and *Mercury* (Macrae *et al.*, 2008 ▶); software used to prepare material for publication: *SHELXL97* and *PLATON* (Spek, 2009 ▶).

## Supplementary Material

Crystal structure: contains datablock(s) I, global. DOI: 10.1107/S160053681103902X/bt5651sup1.cif

Supplementary material file. DOI: 10.1107/S160053681103902X/bt5651Isup2.cdx

Structure factors: contains datablock(s) I. DOI: 10.1107/S160053681103902X/bt5651Isup3.hkl

Supplementary material file. DOI: 10.1107/S160053681103902X/bt5651Isup4.cml

Additional supplementary materials:  crystallographic information; 3D view; checkCIF report

## Figures and Tables

**Table 1:** Hydrogen-bond geometry (Å, °)

*D*—H⋯*A*	*D*—H	H⋯*A*	*D*⋯*A*	*D*—H⋯*A*
N2—H721⋯O22	0.89 (4)	1.81 (4)	2.691 (3)	173 (4)
N2—H722⋯O12^i^	0.99 (4)	1.70 (4)	2.688 (3)	174 (4)
N4—H741⋯O21^ii^	0.94 (3)	1.75 (3)	2.683 (3)	174 (3)
N4—H742⋯O11^iii^	0.98 (4)	1.71 (4)	2.688 (3)	174 (3)
C23—H23⋯F1^iv^	0.95	2.41	3.281 (6)	153
C55—H55⋯F3^iv^	0.95	2.46	3.374 (5)	161
C3—H3*A*⋯O21^iv^	0.99	2.61	3.576 (3)	165
C5—H5*B*⋯O12^v^	0.99	2.60	3.564 (3)	164

Symmetry codes: (i) 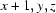; (ii) 
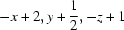
; (iii) 
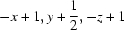
; (iv) 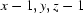; (v) 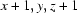.

**Fig. 1.**
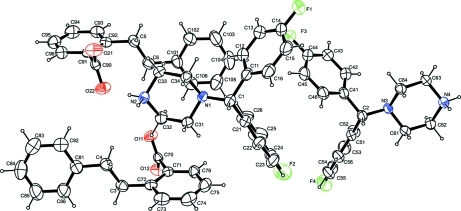
The molecular structure of the title compound, with atom labels and anisotropic displacement ellipsoids (drawn at 50% probability level).

**Fig. 2.**
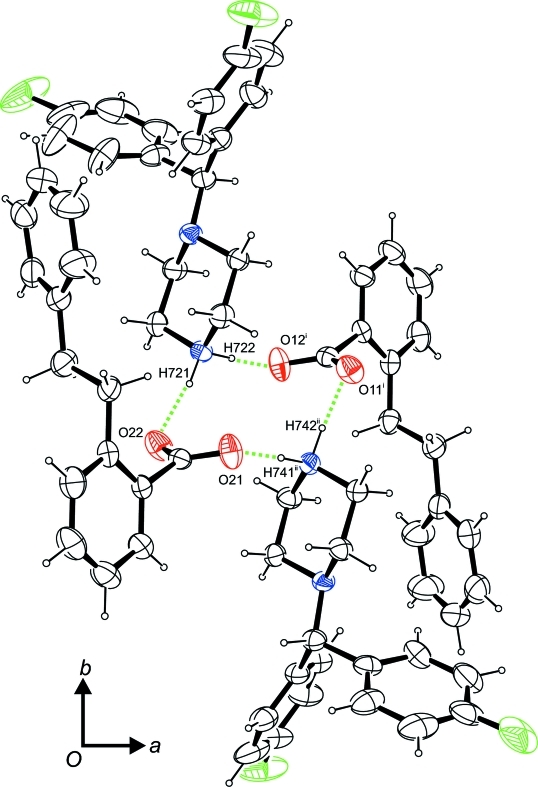
Intermolecular hydrogen bonds, viewed along [0 0 - 1]. Symmetry operators: ix + 1, y, z; ii -x + 2, y - 1/2, -z + 1.

**Fig. 3.**
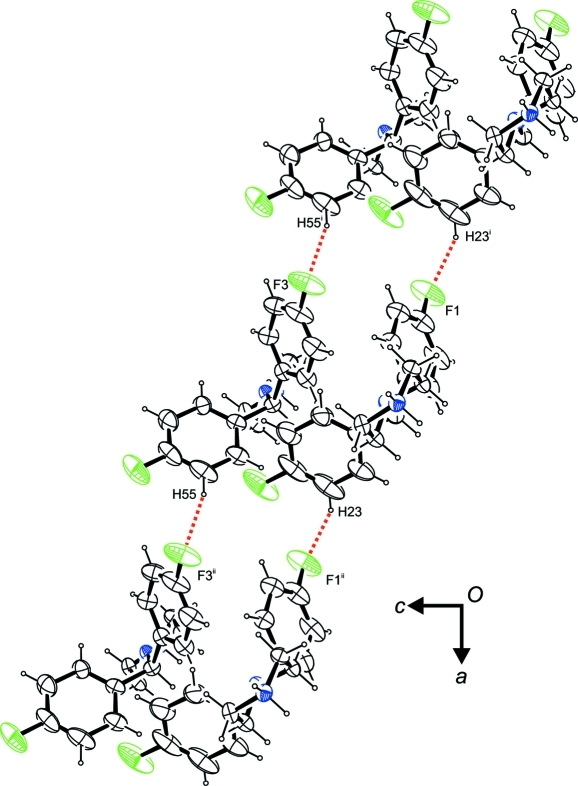
Intermolecular C–H···F contacts, viewed along [0 - 1 0]. Symmetry operators: ix + 1, y, z + 1; iix - 1, y, z - 1.

**Fig. 4.**
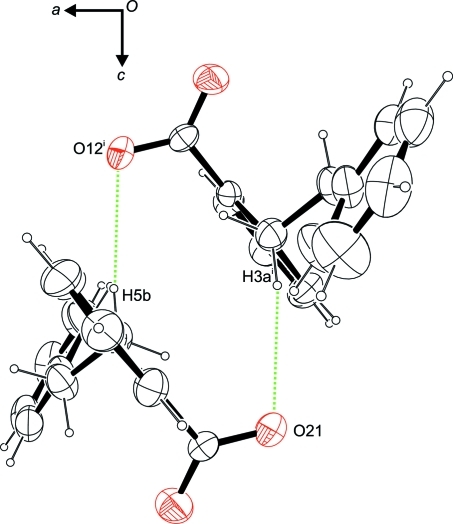
Intermolecular C–H···O contacts, viewed along [0 - 1 0]. Symmetry operator: ix + 1, y, z + 1.

**Fig. 5.**
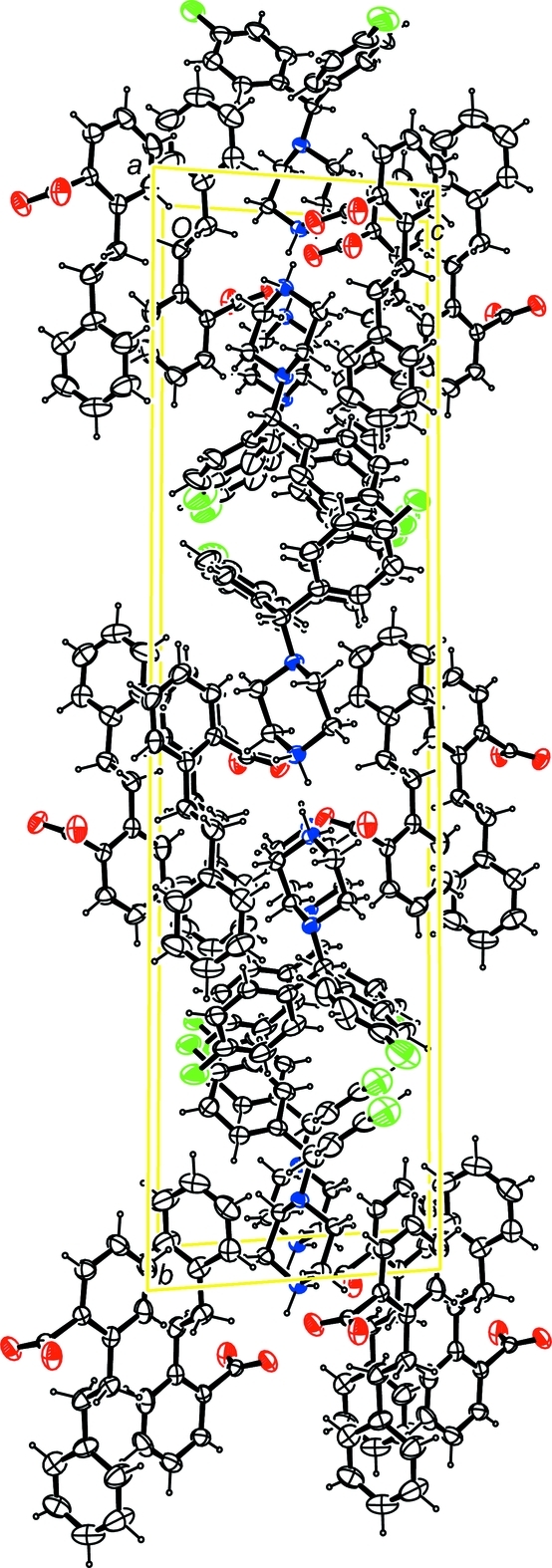
Molecular packing of the title compound, viewed along [-1 0 0] (anisotropic displacement ellipsoids drawn at 50% probability level).

## supplementary crystallographic information

### Comment

4,4'-Difluorobenzhydryl piperazine is an intermediate for the preparation of flunarizine which is a calcium channel blocker. Piperazines are among the most important building blocks in today's drug discovery. They are found in biologically active compounds across a number of different therapeutic areas such as antifungal, antibacterial, antimalarial, antipsychotic, antidepressant and antitumour activity against colon, prostate, breast, lung and leukemia tumors (Brockunier *et al.*, 2004; Bogatcheva *et al.*, 2006). The crystal structures of some related compounds, *viz.*, 2-(2-phenylethyl)benzoic acid (Anilkumar *et al.*, 2005), levocetirizinium dipicrate (Jasinski *et al.*, 2010), cinnarizinium dipicrate (Jasinski *et al.*, 2011), 1-methylpiperazine-1,4- diium dipicrate (Dutkiewicz *et al.*, 2011) and 4-(4-chlorophenyl)-4-hydroxypipe ridinium 2-(2-phenylethyl)benzoate (Fun *et al.*, 2011) have been reported. Recently, we have reported the crystal structure of 4-[bis(4-fluorophenyl) methyl]piperazin-1-ium picrate (Betz *et al.*, 2011). In the course of our studies on the salts of piperazines and in view of the importance of piperazines, the paper reports the crystal and molecular structure of the title salt.

In both cations present in the asymmetric unit protonation occurred on the secondary amine functionality. According to a conformational analysis (Cremer & Pople, 1975), both diazacyclohexane moieties adopt a ^4^*C*_1_*chair* conformation. The least-squares planes defined by the carbon atoms of the *para*-fluorophenyl moieties in the respective cations intersect at angles of 77.21 (19) ° and 78.8 (2) °. The aromatic systems in the anions enclose angles of 69.5 (2) ° and 69.9 (2) °, respectively (Fig. 1).

In the crystal, classical hydrogen bonds as well as C–H···F contacts and C–H···O contacts whose range falls by more than 0.1 Å below the sum of van-der-Waals radii of the atoms participating are observed. The classical hydrogen bonds are apparent between the protonated amine functionality and both oxygen atoms of the carboxylic acid and give rise to the formation of cyclic patterns involving both symmetry-independent cations and anions of the asymmetric unit. While the C–H···F contacts exclusively involve hydrogen atoms in *ortho*-position to the fluorine atoms on the *para*-fluorophenyl moieties and connect only one of the cations with its symmetry-generated equivalents, the C–H···O contacts appear between hydrogen atoms of the methylene groups in the carboxylates and "dimerize" the two independent anions present in the asymmetric unit. In terms of graph-set analysis (Etter *et al.*, 1990; Bernstein *et al.*, 1995), the descriptor for the classical hydrogen bonds is *DDDD* on the unitary level while a *R*^4^_4_(12) descriptor on the quartenary level emphasizes the presence of the cyclic patterns illustrated in Figure 2. For the C–H···F contacts, a *C*^1^_1_(10)*C*^1^_1_(10) descriptor on the unitary level shows the presence of two homodromic chains as depicted in Figure 3 while a *R*^2^_2_(12) descriptor on the binary level is indicative for the cyclization of the two anions present in the asymmetric unit on grounds of C–H···O contacts (Fig. 4). In total, the entities of the title compound are connected to planes parallel to *ac*. The shortest intercentroid distance between two centers of gravity was found at 4.615 (3) Å.

The packing of the title compound in the crystal is shown in Figure 5.

### Experimental

4,4'-Difluorobenzhydryl piperazine was obtained from *R. L*. Fine Chem., Bengaluru, India. 4,4'-Difluorobenzhydryl piperazine (2.88 g, 0.01 mol) was dissolved in 10 ml of methanol and 2-(2-phenylethyl)benzoic acid (2.26 g, 0.01 mol) was also dissolved in 10 ml of methanol. Both the solutions were mixed and stirred in a beaker at 333 K for 60 minutes. The mixture was kept aside for a day at room temperature. The salt formed was filtered & dried in vaccum desiccator over phosphorous pentoxide. X-ray quality crystals of the title compound were obtained from ethanol by slow evaporation.

### Refinement

Carbon-bound H atoms were placed in calculated positions (C—H 0.95 Å for aromatic carbon atoms, C—H 0.99 Å for methylene groups and C—H 1.00 Å for methine groups) and were included in the refinement in the riding model approximation, with *U*(H) set to 1.2*U*_eq_(C). All nitrogen-bound H atoms were located on a difference Fourier map and refined freely. A search for higher or missed symmetry by means of PLATON (Spek, 2009) revealed the presence of a local center of symmetry which is not compatible with the reported space-group symmetry nor with another space group.

### Crystal data

**Table:**

C_17_H_19_F_2_N_2_^+^·C_15_H_13_O_2_^−^	*F*(000) = 1088
*M**_r_* = 514.60	*D*_x_ = 1.250 Mg m^−^^3^
Monoclinic, *P*2_1_	Melting point = 421–423 K
Hall symbol: P 2yb	Mo *K*α radiation, λ = 0.71073 Å
*a* = 8.2330 (2) Å	Cell parameters from 9860 reflections
*b* = 35.5366 (10) Å	θ = 2.7–28.4°
*c* = 10.1505 (3) Å	µ = 0.09 mm^−^^1^
β = 112.925 (1)°	*T* = 200 K
*V* = 2735.19 (13) Å^3^	Block, colourless
*Z* = 4	0.52 × 0.34 × 0.31 mm

### Data collection

**Table:**

Bruker APEXII CCD diffractometer	6401 reflections with *I* > 2σ(*I*)
Radiation source: fine-focus sealed tube	*R*_int_ = 0.033
graphite	θ_max_ = 28.4°, θ_min_ = 2.2°
φ and ω scans	*h* = −10→10
24852 measured reflections	*k* = −47→47
6889 independent reflections	*l* = −13→13

### Refinement

**Table:**

Refinement on *F*^2^	Primary atom site location: structure-invariant direct methods
Least-squares matrix: full	Secondary atom site location: difference Fourier map
*R*[*F*^2^ > 2σ(*F*^2^)] = 0.050	Hydrogen site location: inferred from neighbouring sites
*wR*(*F*^2^) = 0.129	H atoms treated by a mixture of independent and constrained refinement
*S* = 1.07	*w* = 1/[σ^2^(*F*_o_^2^) + (0.0526*P*)^2^ + 1.0497*P*] where *P* = (*F*_o_^2^ + 2*F*_c_^2^)/3
6889 reflections	(Δ/σ)_max_ < 0.001
701 parameters	Δρ_max_ = 0.29 e Å^−^^3^
1 restraint	Δρ_min_ = −0.21 e Å^−^^3^

### Special details

**Table:**

Refinement. Due to the absence of a strong anomalous scatterer, the Flack parameter is meaningless. Thus, Friedel opposites (6158 pairs) have been merged and the item was removed from the CIF.

### Fractional atomic coordinates and isotropic or equivalent isotropic displacement parameters (Å^2^)

**Table:**

	*x*	*y*	*z*	*U*_iso_*/*U*_eq_	
F1	1.4107 (4)	0.32440 (7)	0.8569 (3)	0.0889 (10)	
F2	0.3726 (4)	0.28909 (10)	0.1502 (4)	0.1198 (15)	
N1	1.0316 (3)	0.18603 (6)	0.4512 (2)	0.0314 (5)	
N2	1.0636 (3)	0.10540 (7)	0.4518 (2)	0.0305 (4)	
H721	1.043 (5)	0.0832 (12)	0.482 (4)	0.044 (10)*	
H722	1.118 (5)	0.1010 (11)	0.382 (4)	0.047 (10)*	
C1	1.0484 (4)	0.22471 (8)	0.4080 (3)	0.0355 (6)	
H1	1.1065	0.2243	0.3380	0.043*	
C11	1.1561 (4)	0.24926 (9)	0.5339 (3)	0.0388 (6)	
C12	1.1569 (4)	0.24388 (9)	0.6691 (3)	0.0430 (7)	
H12	1.0970	0.2228	0.6866	0.052*	
C13	1.2436 (5)	0.26877 (10)	0.7796 (4)	0.0517 (8)	
H13	1.2447	0.2651	0.8726	0.062*	
C14	1.3285 (5)	0.29921 (10)	0.7495 (4)	0.0596 (10)	
C15	1.3315 (5)	0.30519 (11)	0.6190 (5)	0.0644 (11)	
H15	1.3920	0.3263	0.6024	0.077*	
C16	1.2453 (4)	0.28013 (10)	0.5099 (4)	0.0498 (8)	
H16	1.2468	0.2840	0.4178	0.060*	
C21	0.8656 (4)	0.24235 (9)	0.3364 (3)	0.0428 (7)	
C22	0.8159 (6)	0.26097 (11)	0.2082 (4)	0.0613 (10)	
H22	0.8960	0.2625	0.1617	0.074*	
C23	0.6517 (7)	0.27755 (12)	0.1455 (6)	0.0815 (17)	
H23	0.6190	0.2909	0.0579	0.098*	
C24	0.5385 (7)	0.27419 (12)	0.2126 (6)	0.0814 (17)	
C25	0.5820 (6)	0.25625 (17)	0.3407 (5)	0.0835 (17)	
H25	0.5007	0.2547	0.3860	0.100*	
C26	0.7489 (5)	0.24032 (14)	0.4030 (4)	0.0634 (11)	
H26	0.7827	0.2279	0.4926	0.076*	
C31	0.9217 (4)	0.16322 (8)	0.3277 (3)	0.0363 (6)	
H31A	0.9803	0.1610	0.2594	0.044*	
H31B	0.8066	0.1758	0.2779	0.044*	
C32	0.8927 (4)	0.12467 (9)	0.3757 (3)	0.0385 (6)	
H32A	0.8286	0.1269	0.4401	0.046*	
H32B	0.8194	0.1096	0.2916	0.046*	
C33	1.1822 (4)	0.12887 (8)	0.5717 (3)	0.0356 (6)	
H33A	1.2989	0.1165	0.6158	0.043*	
H33B	1.1322	0.1312	0.6457	0.043*	
C34	1.2041 (4)	0.16766 (8)	0.5187 (3)	0.0325 (5)	
H34A	1.2818	0.1831	0.6000	0.039*	
H34B	1.2601	0.1655	0.4487	0.039*	
F3	0.9184 (4)	0.30926 (7)	0.8907 (3)	0.0936 (11)	
F4	−0.1354 (4)	0.35140 (9)	0.2007 (4)	0.0943 (10)	
N3	0.5476 (3)	0.44862 (6)	0.4875 (2)	0.0303 (4)	
N4	0.5911 (3)	0.52871 (6)	0.4938 (2)	0.0300 (4)	
H741	0.645 (4)	0.5329 (9)	0.429 (3)	0.027 (7)*	
H742	0.573 (5)	0.5529 (12)	0.533 (4)	0.047 (10)*	
C2	0.5545 (4)	0.40945 (8)	0.4434 (3)	0.0330 (5)	
H2	0.6090	0.4090	0.3709	0.040*	
C41	0.6616 (4)	0.38422 (8)	0.5681 (3)	0.0363 (6)	
C42	0.6641 (5)	0.38961 (9)	0.7039 (4)	0.0444 (7)	
H42	0.6053	0.4107	0.7225	0.053*	
C43	0.7521 (6)	0.36434 (10)	0.8138 (4)	0.0552 (9)	
H43	0.7539	0.3679	0.9071	0.066*	
C44	0.8353 (6)	0.33440 (10)	0.7838 (4)	0.0635 (12)	
C45	0.8376 (5)	0.32788 (11)	0.6516 (5)	0.0617 (11)	
H45	0.8971	0.3067	0.6345	0.074*	
C46	0.7499 (4)	0.35332 (10)	0.5432 (4)	0.0465 (7)	
H46	0.7501	0.3496	0.4506	0.056*	
C51	0.3684 (4)	0.39346 (8)	0.3756 (3)	0.0378 (6)	
C52	0.2507 (4)	0.39973 (10)	0.4405 (4)	0.0462 (7)	
H52	0.2867	0.4143	0.5256	0.055*	
C53	0.0810 (5)	0.38509 (12)	0.3830 (4)	0.0578 (9)	
H53	0.0004	0.3893	0.4277	0.069*	
C54	0.0338 (5)	0.36447 (11)	0.2600 (5)	0.0622 (11)	
C55	0.1451 (6)	0.35709 (11)	0.1942 (5)	0.0675 (12)	
H55	0.1083	0.3421	0.1101	0.081*	
C56	0.3157 (5)	0.37207 (10)	0.2527 (4)	0.0520 (8)	
H56	0.3955	0.3675	0.2076	0.062*	
C61	0.4425 (4)	0.47188 (8)	0.3648 (3)	0.0343 (6)	
H61A	0.3265	0.4599	0.3131	0.041*	
H61B	0.5034	0.4740	0.2981	0.041*	
C62	0.4163 (4)	0.51067 (8)	0.4146 (3)	0.0355 (6)	
H62A	0.3463	0.5263	0.3311	0.043*	
H62B	0.3505	0.5087	0.4778	0.043*	
C63	0.7059 (4)	0.50441 (8)	0.6112 (3)	0.0347 (6)	
H63A	0.6558	0.5021	0.6851	0.042*	
H63B	0.8242	0.5160	0.6562	0.042*	
C64	0.7228 (4)	0.46568 (8)	0.5556 (3)	0.0344 (6)	
H64A	0.7786	0.4677	0.4853	0.041*	
H64B	0.7985	0.4496	0.6356	0.041*	
O11	0.4801 (3)	0.09313 (7)	0.4005 (2)	0.0450 (5)	
O12	0.2001 (3)	0.09716 (8)	0.2522 (2)	0.0479 (6)	
C3	0.4593 (4)	0.05677 (9)	0.0815 (3)	0.0387 (6)	
H3A	0.4231	0.0473	−0.0177	0.046*	
H3B	0.3700	0.0483	0.1179	0.046*	
C4	0.6352 (4)	0.03940 (10)	0.1726 (4)	0.0432 (7)	
H4A	0.6630	0.0462	0.2737	0.052*	
H4B	0.7271	0.0508	0.1451	0.052*	
C70	0.3612 (4)	0.10230 (7)	0.2842 (3)	0.0303 (5)	
C71	0.4142 (3)	0.12085 (8)	0.1735 (3)	0.0286 (5)	
C72	0.4604 (3)	0.09921 (9)	0.0786 (3)	0.0328 (6)	
C73	0.5049 (4)	0.11850 (11)	−0.0232 (3)	0.0468 (8)	
H73	0.5364	0.1044	−0.0892	0.056*	
C74	0.5045 (5)	0.15704 (12)	−0.0306 (4)	0.0528 (9)	
H74	0.5362	0.1692	−0.1005	0.063*	
C75	0.4583 (5)	0.17797 (10)	0.0633 (4)	0.0476 (8)	
H75	0.4574	0.2047	0.0583	0.057*	
C76	0.4130 (4)	0.15991 (9)	0.1651 (3)	0.0384 (6)	
H76	0.3808	0.1744	0.2299	0.046*	
C81	0.6466 (4)	−0.00298 (10)	0.1627 (4)	0.0436 (7)	
C82	0.7804 (4)	−0.02184 (11)	0.2695 (4)	0.0459 (7)	
H82	0.8586	−0.0082	0.3498	0.055*	
C83	0.8023 (5)	−0.06030 (12)	0.2611 (5)	0.0567 (9)	
H83	0.8960	−0.0728	0.3348	0.068*	
C84	0.6894 (6)	−0.08038 (12)	0.1471 (5)	0.0645 (11)	
H84	0.7046	−0.1067	0.1412	0.077*	
C85	0.5545 (7)	−0.06220 (13)	0.0419 (6)	0.0752 (13)	
H85	0.4742	−0.0762	−0.0360	0.090*	
C86	0.5335 (6)	−0.02363 (12)	0.0474 (5)	0.0658 (11)	
H86	0.4414	−0.0113	−0.0280	0.079*	
O21	1.2741 (3)	0.03769 (8)	0.7060 (2)	0.0476 (6)	
O22	0.9933 (3)	0.04102 (7)	0.5589 (2)	0.0474 (6)	
C5	1.0165 (4)	0.07915 (9)	0.8772 (3)	0.0372 (6)	
H5A	1.1055	0.0874	0.8401	0.045*	
H5B	1.0531	0.0889	0.9760	0.045*	
C6	0.8398 (4)	0.09635 (10)	0.7856 (4)	0.0454 (7)	
H6A	0.8111	0.0891	0.6848	0.054*	
H6B	0.7485	0.0852	0.8146	0.054*	
C90	1.1135 (4)	0.03262 (7)	0.6758 (3)	0.0290 (5)	
C91	1.0624 (3)	0.01466 (8)	0.7887 (3)	0.0284 (5)	
C92	1.0155 (3)	0.03688 (8)	0.8817 (3)	0.0318 (5)	
C93	0.9734 (4)	0.01861 (11)	0.9856 (3)	0.0435 (7)	
H93	0.9430	0.0331	1.0512	0.052*	
C94	0.9748 (5)	−0.02027 (12)	0.9953 (4)	0.0505 (8)	
H94	0.9443	−0.0321	1.0665	0.061*	
C95	1.0202 (5)	−0.04179 (10)	0.9028 (4)	0.0479 (8)	
H95	1.0210	−0.0685	0.9093	0.057*	
C96	1.0649 (4)	−0.02419 (9)	0.7995 (3)	0.0394 (6)	
H96	1.0975	−0.0389	0.7356	0.047*	
C101	0.8281 (4)	0.13874 (10)	0.7924 (4)	0.0438 (7)	
C102	0.9426 (6)	0.16027 (12)	0.9050 (5)	0.0647 (11)	
H102	1.0362	0.1484	0.9810	0.078*	
C103	0.9205 (7)	0.19880 (13)	0.9066 (6)	0.0736 (13)	
H103	1.0012	0.2132	0.9828	0.088*	
C104	0.7849 (6)	0.21651 (11)	0.8007 (6)	0.0662 (11)	
H104	0.7693	0.2429	0.8034	0.079*	
C105	0.6722 (5)	0.19542 (11)	0.6909 (5)	0.0555 (9)	
H105	0.5769	0.2075	0.6171	0.067*	
C106	0.6932 (4)	0.15714 (11)	0.6844 (4)	0.0476 (7)	
H106	0.6145	0.1433	0.6052	0.057*	

### Atomic displacement parameters (Å^2^)

**Table:**

	*U*^11^	*U*^22^	*U*^33^	*U*^12^	*U*^13^	*U*^23^
F1	0.096 (2)	0.0387 (12)	0.0827 (17)	−0.0108 (13)	−0.0189 (15)	−0.0037 (12)
F2	0.0812 (19)	0.090 (2)	0.120 (2)	0.0560 (17)	−0.0348 (17)	−0.0352 (19)
N1	0.0293 (11)	0.0271 (11)	0.0342 (11)	0.0013 (9)	0.0084 (9)	0.0027 (8)
N2	0.0326 (11)	0.0275 (11)	0.0334 (11)	0.0002 (9)	0.0150 (9)	0.0045 (9)
C1	0.0360 (14)	0.0322 (14)	0.0376 (14)	0.0025 (11)	0.0136 (11)	0.0054 (11)
C11	0.0347 (14)	0.0318 (14)	0.0455 (15)	0.0053 (11)	0.0107 (12)	0.0092 (11)
C12	0.0468 (17)	0.0325 (15)	0.0447 (16)	−0.0021 (13)	0.0125 (13)	0.0031 (12)
C13	0.056 (2)	0.0381 (17)	0.0487 (18)	0.0050 (15)	0.0068 (15)	−0.0011 (13)
C14	0.056 (2)	0.0283 (16)	0.066 (2)	−0.0024 (15)	−0.0074 (17)	0.0033 (15)
C15	0.051 (2)	0.0377 (18)	0.076 (3)	−0.0085 (15)	−0.0063 (18)	0.0159 (17)
C16	0.0392 (16)	0.0417 (18)	0.062 (2)	−0.0010 (14)	0.0121 (14)	0.0167 (15)
C21	0.0471 (17)	0.0314 (15)	0.0397 (15)	0.0084 (12)	0.0056 (13)	0.0010 (11)
C22	0.061 (2)	0.0430 (19)	0.055 (2)	−0.0063 (16)	−0.0035 (17)	0.0135 (16)
C23	0.073 (3)	0.042 (2)	0.079 (3)	−0.002 (2)	−0.025 (2)	0.018 (2)
C24	0.061 (3)	0.045 (2)	0.088 (3)	0.028 (2)	−0.026 (2)	−0.024 (2)
C25	0.057 (2)	0.113 (4)	0.064 (3)	0.044 (3)	0.006 (2)	−0.025 (3)
C26	0.053 (2)	0.084 (3)	0.0454 (18)	0.032 (2)	0.0104 (15)	−0.0022 (18)
C31	0.0302 (13)	0.0332 (14)	0.0374 (14)	0.0011 (11)	0.0043 (11)	0.0010 (11)
C32	0.0271 (12)	0.0391 (16)	0.0472 (16)	0.0001 (11)	0.0122 (12)	−0.0001 (12)
C33	0.0349 (13)	0.0359 (15)	0.0315 (13)	0.0036 (11)	0.0081 (11)	0.0043 (10)
C34	0.0263 (12)	0.0303 (13)	0.0368 (13)	0.0011 (10)	0.0077 (10)	0.0031 (10)
F3	0.117 (2)	0.0433 (13)	0.0669 (15)	0.0117 (14)	−0.0221 (15)	0.0043 (11)
F4	0.0596 (14)	0.0745 (18)	0.112 (2)	−0.0370 (13)	−0.0065 (14)	−0.0032 (16)
N3	0.0278 (11)	0.0243 (10)	0.0350 (11)	−0.0013 (8)	0.0081 (9)	−0.0030 (8)
N4	0.0299 (11)	0.0287 (11)	0.0342 (11)	−0.0016 (9)	0.0155 (9)	−0.0035 (8)
C2	0.0388 (14)	0.0272 (13)	0.0341 (13)	−0.0006 (11)	0.0153 (11)	−0.0034 (10)
C41	0.0360 (14)	0.0269 (13)	0.0436 (15)	−0.0009 (11)	0.0129 (12)	−0.0039 (11)
C42	0.0516 (18)	0.0336 (16)	0.0454 (16)	0.0010 (13)	0.0159 (14)	−0.0008 (12)
C43	0.069 (2)	0.0392 (18)	0.0427 (17)	−0.0043 (16)	0.0053 (16)	0.0003 (13)
C44	0.069 (2)	0.0320 (17)	0.057 (2)	−0.0004 (16)	−0.0104 (18)	0.0013 (14)
C45	0.058 (2)	0.0330 (17)	0.071 (2)	0.0139 (15)	−0.0001 (18)	−0.0100 (16)
C46	0.0436 (17)	0.0359 (16)	0.0530 (18)	0.0030 (13)	0.0111 (14)	−0.0089 (13)
C51	0.0432 (15)	0.0249 (13)	0.0375 (14)	−0.0049 (11)	0.0073 (12)	−0.0025 (10)
C52	0.0448 (17)	0.0463 (17)	0.0464 (17)	−0.0168 (14)	0.0163 (14)	−0.0050 (13)
C53	0.0496 (19)	0.054 (2)	0.065 (2)	−0.0190 (16)	0.0168 (17)	0.0047 (17)
C54	0.049 (2)	0.0357 (17)	0.075 (3)	−0.0171 (15)	−0.0046 (18)	0.0060 (16)
C55	0.067 (3)	0.0378 (18)	0.065 (2)	−0.0010 (17)	−0.0110 (19)	−0.0186 (16)
C56	0.057 (2)	0.0368 (16)	0.0480 (18)	0.0038 (14)	0.0049 (15)	−0.0126 (14)
C61	0.0306 (13)	0.0311 (14)	0.0347 (13)	−0.0041 (11)	0.0056 (10)	−0.0033 (10)
C62	0.0271 (12)	0.0307 (14)	0.0467 (15)	0.0003 (10)	0.0122 (11)	−0.0012 (11)
C63	0.0355 (13)	0.0293 (13)	0.0345 (13)	−0.0031 (11)	0.0084 (11)	−0.0036 (10)
C64	0.0255 (12)	0.0326 (14)	0.0402 (14)	−0.0008 (10)	0.0074 (10)	−0.0027 (11)
O11	0.0446 (12)	0.0491 (13)	0.0376 (11)	−0.0035 (10)	0.0119 (9)	0.0152 (9)
O12	0.0352 (11)	0.0733 (17)	0.0412 (11)	−0.0098 (11)	0.0214 (9)	0.0006 (11)
C3	0.0359 (14)	0.0436 (17)	0.0376 (14)	0.0001 (12)	0.0155 (12)	−0.0065 (12)
C4	0.0326 (14)	0.0419 (16)	0.0501 (17)	−0.0010 (12)	0.0106 (12)	−0.0086 (13)
C70	0.0397 (14)	0.0266 (12)	0.0292 (12)	−0.0022 (10)	0.0185 (10)	−0.0040 (9)
C71	0.0241 (11)	0.0342 (13)	0.0268 (11)	−0.0040 (10)	0.0092 (9)	0.0023 (9)
C72	0.0271 (12)	0.0427 (16)	0.0286 (12)	0.0007 (11)	0.0108 (10)	0.0007 (10)
C73	0.0440 (16)	0.066 (2)	0.0367 (15)	−0.0039 (15)	0.0229 (13)	−0.0004 (14)
C74	0.0529 (19)	0.064 (2)	0.0466 (18)	−0.0066 (17)	0.0246 (15)	0.0187 (16)
C75	0.0478 (18)	0.0401 (17)	0.0538 (19)	−0.0073 (14)	0.0187 (15)	0.0123 (14)
C76	0.0427 (15)	0.0318 (14)	0.0426 (15)	−0.0044 (12)	0.0185 (12)	0.0010 (11)
C81	0.0313 (14)	0.0442 (17)	0.0551 (18)	−0.0005 (13)	0.0166 (13)	−0.0083 (14)
C82	0.0386 (16)	0.055 (2)	0.0481 (17)	−0.0031 (14)	0.0215 (13)	0.0008 (14)
C83	0.052 (2)	0.059 (2)	0.069 (2)	0.0083 (18)	0.0354 (18)	0.0204 (19)
C84	0.069 (3)	0.0378 (19)	0.099 (3)	0.0017 (18)	0.047 (2)	0.0075 (19)
C85	0.076 (3)	0.045 (2)	0.089 (3)	−0.007 (2)	0.014 (2)	−0.018 (2)
C86	0.055 (2)	0.045 (2)	0.075 (3)	0.0003 (17)	0.0012 (19)	−0.0105 (18)
O21	0.0380 (11)	0.0722 (17)	0.0379 (11)	−0.0064 (11)	0.0206 (9)	0.0020 (10)
O22	0.0448 (12)	0.0552 (14)	0.0382 (11)	−0.0088 (11)	0.0118 (9)	0.0152 (10)
C5	0.0346 (14)	0.0375 (15)	0.0431 (15)	−0.0010 (11)	0.0190 (12)	−0.0079 (12)
C6	0.0341 (14)	0.0447 (18)	0.0513 (18)	−0.0007 (13)	0.0100 (13)	−0.0109 (14)
C90	0.0355 (13)	0.0249 (12)	0.0297 (12)	−0.0029 (10)	0.0162 (10)	−0.0013 (9)
C91	0.0232 (11)	0.0329 (13)	0.0284 (12)	−0.0018 (9)	0.0094 (9)	0.0014 (9)
C92	0.0266 (12)	0.0402 (15)	0.0299 (12)	−0.0030 (11)	0.0123 (10)	−0.0012 (10)
C93	0.0464 (17)	0.057 (2)	0.0341 (14)	−0.0012 (15)	0.0226 (13)	0.0005 (13)
C94	0.056 (2)	0.060 (2)	0.0409 (16)	−0.0091 (17)	0.0245 (14)	0.0137 (14)
C95	0.0498 (19)	0.0427 (18)	0.0494 (18)	−0.0071 (14)	0.0174 (15)	0.0115 (13)
C96	0.0395 (15)	0.0380 (16)	0.0436 (15)	−0.0032 (12)	0.0194 (12)	0.0009 (12)
C101	0.0344 (14)	0.0357 (15)	0.0619 (19)	−0.0020 (12)	0.0195 (14)	−0.0098 (14)
C102	0.050 (2)	0.049 (2)	0.072 (3)	−0.0002 (17)	−0.0006 (18)	−0.0118 (19)
C103	0.071 (3)	0.047 (2)	0.091 (3)	−0.011 (2)	0.019 (2)	−0.023 (2)
C104	0.067 (3)	0.0350 (19)	0.109 (4)	−0.0043 (17)	0.047 (3)	−0.0024 (19)
C105	0.0471 (18)	0.049 (2)	0.077 (2)	0.0017 (16)	0.0314 (18)	0.0175 (18)
C106	0.0366 (15)	0.054 (2)	0.0567 (19)	−0.0030 (14)	0.0226 (14)	−0.0064 (15)

### Geometric parameters (Å, °)

**Table:**

F1—C14	1.369 (4)	C56—H56	0.9500
F2—C24	1.369 (5)	C61—C62	1.512 (4)
N1—C1	1.466 (4)	C61—H61A	0.9900
N1—C34	1.468 (3)	C61—H61B	0.9900
N1—C31	1.471 (3)	C62—H62A	0.9900
N2—C32	1.483 (4)	C62—H62B	0.9900
N2—C33	1.485 (4)	C63—C64	1.514 (4)
N2—H721	0.89 (4)	C63—H63A	0.9900
N2—H722	0.99 (4)	C63—H63B	0.9900
C1—C11	1.515 (4)	C64—H64A	0.9900
C1—C21	1.528 (4)	C64—H64B	0.9900
C1—H1	1.0000	O11—C70	1.248 (3)
C11—C12	1.383 (5)	O12—C70	1.250 (3)
C11—C16	1.393 (4)	C3—C72	1.508 (4)
C12—C13	1.388 (5)	C3—C4	1.513 (4)
C12—H12	0.9500	C3—H3A	0.9900
C13—C14	1.385 (6)	C3—H3B	0.9900
C13—H13	0.9500	C4—C81	1.515 (5)
C14—C15	1.351 (6)	C4—H4A	0.9900
C15—C16	1.382 (6)	C4—H4B	0.9900
C15—H15	0.9500	C70—C71	1.506 (3)
C16—H16	0.9500	C71—C76	1.391 (4)
C21—C22	1.373 (5)	C71—C72	1.396 (4)
C21—C26	1.375 (6)	C72—C73	1.402 (4)
C22—C23	1.382 (6)	C73—C74	1.372 (6)
C22—H22	0.9500	C73—H73	0.9500
C23—C24	1.358 (8)	C74—C75	1.375 (6)
C23—H23	0.9500	C74—H74	0.9500
C24—C25	1.365 (8)	C75—C76	1.385 (4)
C25—C26	1.390 (5)	C75—H75	0.9500
C25—H25	0.9500	C76—H76	0.9500
C26—H26	0.9500	C81—C82	1.381 (5)
C31—C32	1.504 (4)	C81—C86	1.388 (5)
C31—H31A	0.9900	C82—C83	1.386 (6)
C31—H31B	0.9900	C82—H82	0.9500
C32—H32A	0.9900	C83—C84	1.369 (6)
C32—H32B	0.9900	C83—H83	0.9500
C33—C34	1.515 (4)	C84—C85	1.366 (7)
C33—H33A	0.9900	C84—H84	0.9500
C33—H33B	0.9900	C85—C86	1.386 (6)
C34—H34A	0.9900	C85—H85	0.9500
C34—H34B	0.9900	C86—H86	0.9500
F3—C44	1.365 (4)	O21—C90	1.249 (3)
F4—C54	1.366 (4)	O22—C90	1.250 (3)
N3—C61	1.464 (3)	C5—C92	1.503 (4)
N3—C64	1.466 (3)	C5—C6	1.517 (4)
N3—C2	1.470 (3)	C5—H5A	0.9900
N4—C63	1.477 (4)	C5—H5B	0.9900
N4—C62	1.492 (3)	C6—C101	1.513 (5)
N4—H741	0.94 (3)	C6—H6A	0.9900
N4—H742	0.98 (4)	C6—H6B	0.9900
C2—C41	1.521 (4)	C90—C91	1.508 (3)
C2—C51	1.524 (4)	C91—C96	1.384 (4)
C2—H2	1.0000	C91—C92	1.396 (4)
C41—C42	1.384 (5)	C92—C93	1.393 (4)
C41—C46	1.393 (4)	C93—C94	1.385 (5)
C42—C43	1.395 (5)	C93—H93	0.9500
C42—H42	0.9500	C94—C95	1.371 (6)
C43—C44	1.362 (6)	C94—H94	0.9500
C43—H43	0.9500	C95—C96	1.388 (4)
C44—C45	1.370 (7)	C95—H95	0.9500
C45—C46	1.389 (5)	C96—H96	0.9500
C45—H45	0.9500	C101—C106	1.383 (5)
C46—H46	0.9500	C101—C102	1.393 (5)
C51—C56	1.378 (4)	C102—C103	1.383 (6)
C51—C52	1.386 (5)	C102—H102	0.9500
C52—C53	1.388 (5)	C103—C104	1.365 (7)
C52—H52	0.9500	C103—H103	0.9500
C53—C54	1.368 (6)	C104—C105	1.363 (6)
C53—H53	0.9500	C104—H104	0.9500
C54—C55	1.353 (7)	C105—C106	1.376 (6)
C55—C56	1.399 (6)	C105—H105	0.9500
C55—H55	0.9500	C106—H106	0.9500
			
C1—N1—C34	111.7 (2)	N3—C61—C62	110.1 (2)
C1—N1—C31	111.1 (2)	N3—C61—H61A	109.6
C34—N1—C31	108.4 (2)	C62—C61—H61A	109.6
C32—N2—C33	110.8 (2)	N3—C61—H61B	109.6
C32—N2—H721	109 (2)	C62—C61—H61B	109.6
C33—N2—H721	111 (2)	H61A—C61—H61B	108.1
C32—N2—H722	108 (2)	N4—C62—C61	109.8 (2)
C33—N2—H722	110 (2)	N4—C62—H62A	109.7
H721—N2—H722	108 (3)	C61—C62—H62A	109.7
N1—C1—C11	112.4 (2)	N4—C62—H62B	109.7
N1—C1—C21	109.7 (2)	C61—C62—H62B	109.7
C11—C1—C21	108.0 (2)	H62A—C62—H62B	108.2
N1—C1—H1	108.9	N4—C63—C64	110.7 (2)
C11—C1—H1	108.9	N4—C63—H63A	109.5
C21—C1—H1	108.9	C64—C63—H63A	109.5
C12—C11—C16	118.9 (3)	N4—C63—H63B	109.5
C12—C11—C1	122.1 (3)	C64—C63—H63B	109.5
C16—C11—C1	118.8 (3)	H63A—C63—H63B	108.1
C11—C12—C13	121.0 (3)	N3—C64—C63	109.6 (2)
C11—C12—H12	119.5	N3—C64—H64A	109.7
C13—C12—H12	119.5	C63—C64—H64A	109.7
C14—C13—C12	117.7 (4)	N3—C64—H64B	109.7
C14—C13—H13	121.2	C63—C64—H64B	109.7
C12—C13—H13	121.2	H64A—C64—H64B	108.2
C15—C14—F1	119.1 (4)	C72—C3—C4	114.1 (3)
C15—C14—C13	122.8 (3)	C72—C3—H3A	108.7
F1—C14—C13	118.1 (4)	C4—C3—H3A	108.7
C14—C15—C16	118.9 (4)	C72—C3—H3B	108.7
C14—C15—H15	120.5	C4—C3—H3B	108.7
C16—C15—H15	120.5	H3A—C3—H3B	107.6
C15—C16—C11	120.6 (4)	C3—C4—C81	115.7 (3)
C15—C16—H16	119.7	C3—C4—H4A	108.3
C11—C16—H16	119.7	C81—C4—H4A	108.3
C22—C21—C26	119.0 (4)	C3—C4—H4B	108.3
C22—C21—C1	121.5 (4)	C81—C4—H4B	108.3
C26—C21—C1	119.5 (3)	H4A—C4—H4B	107.4
C21—C22—C23	121.2 (5)	O11—C70—O12	124.7 (3)
C21—C22—H22	119.4	O11—C70—C71	118.1 (2)
C23—C22—H22	119.4	O12—C70—C71	117.2 (2)
C24—C23—C22	118.1 (4)	C76—C71—C72	120.3 (2)
C24—C23—H23	120.9	C76—C71—C70	119.1 (2)
C22—C23—H23	120.9	C72—C71—C70	120.6 (2)
C23—C24—C25	122.9 (4)	C71—C72—C73	117.3 (3)
C23—C24—F2	119.3 (5)	C71—C72—C3	122.1 (2)
C25—C24—F2	117.8 (6)	C73—C72—C3	120.5 (3)
C24—C25—C26	118.0 (5)	C74—C73—C72	122.2 (3)
C24—C25—H25	121.0	C74—C73—H73	118.9
C26—C25—H25	121.0	C72—C73—H73	118.9
C21—C26—C25	120.7 (4)	C73—C74—C75	119.8 (3)
C21—C26—H26	119.6	C73—C74—H74	120.1
C25—C26—H26	119.6	C75—C74—H74	120.1
N1—C31—C32	110.4 (2)	C74—C75—C76	119.6 (3)
N1—C31—H31A	109.6	C74—C75—H75	120.2
C32—C31—H31A	109.6	C76—C75—H75	120.2
N1—C31—H31B	109.6	C75—C76—C71	120.7 (3)
C32—C31—H31B	109.6	C75—C76—H76	119.6
H31A—C31—H31B	108.1	C71—C76—H76	119.6
N2—C32—C31	110.5 (2)	C82—C81—C86	118.3 (3)
N2—C32—H32A	109.5	C82—C81—C4	118.8 (3)
C31—C32—H32A	109.5	C86—C81—C4	122.9 (3)
N2—C32—H32B	109.5	C81—C82—C83	120.9 (3)
C31—C32—H32B	109.5	C81—C82—H82	119.6
H32A—C32—H32B	108.1	C83—C82—H82	119.6
N2—C33—C34	110.5 (2)	C84—C83—C82	120.3 (4)
N2—C33—H33A	109.5	C84—C83—H83	119.9
C34—C33—H33A	109.5	C82—C83—H83	119.9
N2—C33—H33B	109.5	C85—C84—C83	119.5 (4)
C34—C33—H33B	109.5	C85—C84—H84	120.3
H33A—C33—H33B	108.1	C83—C84—H84	120.3
N1—C34—C33	110.1 (2)	C84—C85—C86	120.8 (4)
N1—C34—H34A	109.6	C84—C85—H85	119.6
C33—C34—H34A	109.6	C86—C85—H85	119.6
N1—C34—H34B	109.6	C85—C86—C81	120.3 (4)
C33—C34—H34B	109.6	C85—C86—H86	119.9
H34A—C34—H34B	108.2	C81—C86—H86	119.9
C61—N3—C64	108.3 (2)	C92—C5—C6	114.0 (3)
C61—N3—C2	110.8 (2)	C92—C5—H5A	108.8
C64—N3—C2	112.8 (2)	C6—C5—H5A	108.8
C63—N4—C62	111.3 (2)	C92—C5—H5B	108.8
C63—N4—H741	109 (2)	C6—C5—H5B	108.8
C62—N4—H741	108.0 (19)	H5A—C5—H5B	107.7
C63—N4—H742	110 (2)	C101—C6—C5	115.9 (3)
C62—N4—H742	109 (2)	C101—C6—H6A	108.3
H741—N4—H742	109 (3)	C5—C6—H6A	108.3
N3—C2—C41	112.5 (2)	C101—C6—H6B	108.3
N3—C2—C51	109.8 (2)	C5—C6—H6B	108.3
C41—C2—C51	108.1 (2)	H6A—C6—H6B	107.4
N3—C2—H2	108.8	O21—C90—O22	124.7 (2)
C41—C2—H2	108.8	O21—C90—C91	117.2 (2)
C51—C2—H2	108.8	O22—C90—C91	118.1 (2)
C42—C41—C46	118.9 (3)	C96—C91—C92	120.7 (2)
C42—C41—C2	121.7 (3)	C96—C91—C90	118.8 (2)
C46—C41—C2	119.2 (3)	C92—C91—C90	120.5 (2)
C41—C42—C43	120.6 (3)	C93—C92—C91	117.7 (3)
C41—C42—H42	119.7	C93—C92—C5	119.6 (3)
C43—C42—H42	119.7	C91—C92—C5	122.6 (2)
C44—C43—C42	118.3 (4)	C94—C93—C92	121.4 (3)
C44—C43—H43	120.9	C94—C93—H93	119.3
C42—C43—H43	120.9	C92—C93—H93	119.3
C43—C44—F3	118.3 (4)	C95—C94—C93	120.3 (3)
C43—C44—C45	123.4 (3)	C95—C94—H94	119.8
F3—C44—C45	118.3 (4)	C93—C94—H94	119.8
C44—C45—C46	117.7 (3)	C94—C95—C96	119.3 (3)
C44—C45—H45	121.2	C94—C95—H95	120.4
C46—C45—H45	121.2	C96—C95—H95	120.4
C45—C46—C41	121.1 (3)	C91—C96—C95	120.6 (3)
C45—C46—H46	119.5	C91—C96—H96	119.7
C41—C46—H46	119.5	C95—C96—H96	119.7
C56—C51—C52	119.1 (3)	C106—C101—C102	117.9 (3)
C56—C51—C2	121.4 (3)	C106—C101—C6	118.7 (3)
C52—C51—C2	119.4 (3)	C102—C101—C6	123.3 (3)
C51—C52—C53	121.1 (3)	C103—C102—C101	120.2 (4)
C51—C52—H52	119.5	C103—C102—H102	119.9
C53—C52—H52	119.5	C101—C102—H102	119.9
C54—C53—C52	117.7 (4)	C104—C103—C102	121.2 (4)
C54—C53—H53	121.1	C104—C103—H103	119.4
C52—C53—H53	121.1	C102—C103—H103	119.4
C55—C54—F4	119.2 (4)	C105—C104—C103	118.5 (4)
C55—C54—C53	123.3 (4)	C105—C104—H104	120.7
F4—C54—C53	117.5 (5)	C103—C104—H104	120.7
C54—C55—C56	118.5 (4)	C104—C105—C106	121.6 (4)
C54—C55—H55	120.7	C104—C105—H105	119.2
C56—C55—H55	120.7	C106—C105—H105	119.2
C51—C56—C55	120.3 (4)	C105—C106—C101	120.4 (3)
C51—C56—H56	119.9	C105—C106—H106	119.8
C55—C56—H56	119.9	C101—C106—H106	119.8
			
C34—N1—C1—C11	−60.7 (3)	C53—C54—C55—C56	−1.7 (6)
C31—N1—C1—C11	178.1 (2)	C52—C51—C56—C55	0.4 (5)
C34—N1—C1—C21	179.2 (2)	C2—C51—C56—C55	178.7 (3)
C31—N1—C1—C21	58.0 (3)	C54—C55—C56—C51	0.7 (6)
N1—C1—C11—C12	−32.0 (4)	C64—N3—C61—C62	−63.3 (3)
C21—C1—C11—C12	89.1 (3)	C2—N3—C61—C62	172.4 (2)
N1—C1—C11—C16	153.4 (3)	C63—N4—C62—C61	−53.9 (3)
C21—C1—C11—C16	−85.5 (3)	N3—C61—C62—N4	58.7 (3)
C16—C11—C12—C13	0.4 (5)	C62—N4—C63—C64	54.1 (3)
C1—C11—C12—C13	−174.1 (3)	C61—N3—C64—C63	62.6 (3)
C11—C12—C13—C14	0.3 (5)	C2—N3—C64—C63	−174.3 (2)
C12—C13—C14—C15	−0.8 (6)	N4—C63—C64—N3	−58.5 (3)
C12—C13—C14—F1	178.4 (3)	C72—C3—C4—C81	−172.1 (3)
F1—C14—C15—C16	−178.7 (4)	O11—C70—C71—C76	−95.5 (3)
C13—C14—C15—C16	0.5 (6)	O12—C70—C71—C76	84.6 (3)
C14—C15—C16—C11	0.2 (6)	O11—C70—C71—C72	85.8 (3)
C12—C11—C16—C15	−0.7 (5)	O12—C70—C71—C72	−94.1 (3)
C1—C11—C16—C15	174.0 (3)	C76—C71—C72—C73	0.2 (4)
N1—C1—C21—C22	−131.5 (3)	C70—C71—C72—C73	178.8 (3)
C11—C1—C21—C22	105.8 (4)	C76—C71—C72—C3	−178.5 (3)
N1—C1—C21—C26	50.0 (4)	C70—C71—C72—C3	0.1 (4)
C11—C1—C21—C26	−72.8 (4)	C4—C3—C72—C71	−92.8 (3)
C26—C21—C22—C23	0.0 (6)	C4—C3—C72—C73	88.6 (3)
C1—C21—C22—C23	−178.6 (3)	C71—C72—C73—C74	0.2 (5)
C21—C22—C23—C24	−1.5 (6)	C3—C72—C73—C74	178.9 (3)
C22—C23—C24—C25	2.1 (7)	C72—C73—C74—C75	−0.4 (6)
C22—C23—C24—F2	−177.5 (4)	C73—C74—C75—C76	0.2 (5)
C23—C24—C25—C26	−1.1 (8)	C74—C75—C76—C71	0.1 (5)
F2—C24—C25—C26	178.6 (4)	C72—C71—C76—C75	−0.3 (5)
C22—C21—C26—C25	1.1 (6)	C70—C71—C76—C75	−179.0 (3)
C1—C21—C26—C25	179.7 (4)	C3—C4—C81—C82	−161.7 (3)
C24—C25—C26—C21	−0.6 (7)	C3—C4—C81—C86	21.6 (5)
C1—N1—C31—C32	−175.0 (2)	C86—C81—C82—C83	0.6 (5)
C34—N1—C31—C32	61.9 (3)	C4—C81—C82—C83	−176.3 (3)
C33—N2—C32—C31	54.6 (3)	C81—C82—C83—C84	−0.9 (5)
N1—C31—C32—N2	−58.7 (3)	C82—C83—C84—C85	−0.2 (6)
C32—N2—C33—C34	−54.4 (3)	C83—C84—C85—C86	1.7 (8)
C1—N1—C34—C33	175.7 (2)	C84—C85—C86—C81	−2.0 (8)
C31—N1—C34—C33	−61.5 (3)	C82—C81—C86—C85	0.8 (6)
N2—C33—C34—N1	58.4 (3)	C4—C81—C86—C85	177.6 (4)
C61—N3—C2—C41	−179.6 (2)	C92—C5—C6—C101	172.6 (3)
C64—N3—C2—C41	58.7 (3)	O21—C90—C91—C96	−84.3 (3)
C61—N3—C2—C51	−59.1 (3)	O22—C90—C91—C96	95.1 (3)
C64—N3—C2—C51	179.2 (2)	O21—C90—C91—C92	94.9 (3)
N3—C2—C41—C42	34.3 (4)	O22—C90—C91—C92	−85.7 (3)
C51—C2—C41—C42	−87.2 (3)	C96—C91—C92—C93	0.6 (4)
N3—C2—C41—C46	−150.2 (3)	C90—C91—C92—C93	−178.6 (3)
C51—C2—C41—C46	88.4 (3)	C96—C91—C92—C5	178.0 (3)
C46—C41—C42—C43	−0.6 (5)	C90—C91—C92—C5	−1.3 (4)
C2—C41—C42—C43	174.9 (3)	C6—C5—C92—C93	−89.2 (3)
C41—C42—C43—C44	0.1 (6)	C6—C5—C92—C91	93.4 (3)
C42—C43—C44—F3	−178.7 (4)	C91—C92—C93—C94	−1.1 (5)
C42—C43—C44—C45	0.2 (7)	C5—C92—C93—C94	−178.5 (3)
C43—C44—C45—C46	0.0 (7)	C92—C93—C94—C95	0.7 (6)
F3—C44—C45—C46	178.9 (4)	C93—C94—C95—C96	0.1 (5)
C44—C45—C46—C41	−0.5 (6)	C92—C91—C96—C95	0.2 (4)
C42—C41—C46—C45	0.8 (5)	C90—C91—C96—C95	179.5 (3)
C2—C41—C46—C45	−174.9 (3)	C94—C95—C96—C91	−0.6 (5)
N3—C2—C51—C56	136.8 (3)	C5—C6—C101—C106	161.6 (3)
C41—C2—C51—C56	−100.1 (3)	C5—C6—C101—C102	−20.9 (5)
N3—C2—C51—C52	−44.8 (4)	C106—C101—C102—C103	−0.3 (7)
C41—C2—C51—C52	78.3 (3)	C6—C101—C102—C103	−177.8 (4)
C56—C51—C52—C53	−0.6 (5)	C101—C102—C103—C104	1.5 (8)
C2—C51—C52—C53	−179.0 (3)	C102—C103—C104—C105	−1.1 (8)
C51—C52—C53—C54	−0.3 (6)	C103—C104—C105—C106	−0.5 (6)
C52—C53—C54—C55	1.5 (6)	C104—C105—C106—C101	1.7 (6)
C52—C53—C54—F4	−177.4 (3)	C102—C101—C106—C105	−1.3 (5)
F4—C54—C55—C56	177.2 (4)	C6—C101—C106—C105	176.3 (3)

### Hydrogen-bond geometry (Å, °)

**Table:**

*D*—H···*A*	*D*—H	H···*A*	*D*···*A*	*D*—H···*A*
N2—H721···O22	0.89 (4)	1.81 (4)	2.691 (3)	173 (4)
N2—H722···O12^i^	0.99 (4)	1.70 (4)	2.688 (3)	174 (4)
N4—H741···O21^ii^	0.94 (3)	1.75 (3)	2.683 (3)	174 (3)
N4—H742···O11^iii^	0.98 (4)	1.71 (4)	2.688 (3)	174 (3)
C23—H23···F1^iv^	0.95	2.41	3.281 (6)	153
C55—H55···F3^iv^	0.95	2.46	3.374 (5)	161
C3—H3A···O21^iv^	0.99	2.61	3.576 (3)	165
C5—H5B···O12^v^	0.99	2.60	3.564 (3)	164

Symmetry codes: (i) *x*+1, *y*, *z*; (ii) −*x*+2, *y*+1/2, −*z*+1; (iii) −*x*+1, *y*+1/2, −*z*+1; (iv) *x*−1, *y*, *z*−1; (v) *x*+1, *y*, *z*+1.
